# POLICIES TO OUTCOMES: MULTILEVEL CONSIDERATIONS FOR THE ASSISTED LIVING WORKFORCE

**DOI:** 10.1093/geroni/igad104.1096

**Published:** 2023-12-21

**Authors:** Sarah Dys, Cassandra Hua, Kezia Scales

## Abstract

Assisted living (AL) staff mix, levels, training requirements, regulatory enforcement, and career opportunities vary widely across license types, companies, and states. ALs represent the largest care providers for people living with dementia across the U.S. In most states, ALs must have enough qualified, trained staff to sufficiently meet the needs of their resident populations. While the use of interventions to address appropriate staffing levels and workforce retention is increasing, little is known about how the variation in the AL workforce relates to resident and staff experiences or manifests from policies governing these settings. This symposium uses a socioecological framework to examine AL-specific workforce variation. Presentations focus on national variation in staff training requirements, workforce recruitment and retention, trends in staffing levels, how staffing levels can relate to resident outcomes, and direct care staff experiences with stress and coping in long-term care communities. Findings emphasize similarities and differences in workforce composition and experience between states and across different types of facilities. From national comparison of state regulations to facility contexts to individual resident and staff experiences, this panel embraces multilevel contextual understanding of how policies, practices, and outcomes can intersect across AL and residential care environments. This is an Assisted Living Interest Group Sponsored Symposium.

